# Establishing Priorities for Clinical Education Research: Exploring the Views of UK Professional and Public Stakeholders

**DOI:** 10.1111/tct.70144

**Published:** 2025-06-30

**Authors:** Bryan Burford, Peter Yeates, Anna Harvey Bluemel, Sophie Park, John Sandars, Cecily Henry, Clare Corness‐Parr, Richard Conn, Tom Gale, Tim O'Brien, Rikki Goddard‐Fuller, Gill Vance, Janice Ellis

**Affiliations:** ^1^ Newcastle University Newcastle Upon Tyne UK; ^2^ Keele University Stoke on Trent UK; ^3^ University of Oxford Oxford UK; ^4^ Edge Hill University Ormskirk UK; ^5^ University College London London UK; ^6^ Ulster University Coleraine UK; ^7^ University of Plymouth Plymouth UK; ^8^ Christie Education Manchester UK

**Keywords:** health professions education, patient involvement, priority setting, research funding, research impact, research priorities

## Abstract

**Introduction:**

High quality clinical education research is required to ensure optimal education and training of healthcare professionals. Such research should address stakeholder needs and have a clear route to achieving benefit. We conducted the first UK‐wide priority setting exercise for clinical education research to identify research priorities and how they are determined.

**Methods:**

We used a two‐stage process, derived from similar studies, to identify the research priorities of stakeholders including funders, regulators, educators and public representatives. Stage one consisted of two rounds of online surveys, gathering free‐text suggestions of priorities and rating the resulting statements. A public engagement author advised on wording. Stage two used a stakeholder workshop to discuss principles and processes for operationalising priorities and maximising impact.

**Results:**

Round 1 survey respondents (*n* = 256) provided 1819 suggestions, from which content analysis synthesised 46 statements describing disparate research priorities. Distributions of ratings in Round 2 (*n* = 199) indicated that all were perceived as important by most respondents, although professionals and members of the public differed in their rating of some items. Workshop participants (*n* = 70) considered priorities to be dynamic and contextually dependent and linked to expected impact.

**Discussion:**

The study identifies broad priorities for clinical education research, but recognises that simple prioritisation is insufficient, and develops understanding of how priorities arise, including differences between stakeholder groups, and changes over time. Recognising an integrated ‘system of impact’ may maximise opportunities for stakeholders—researchers, policy actors, knowledge users and funders—to effectively communicate and optimise research impact in the short and longer term.

## 1 Introduction

High‐quality education and training is vital for safe and effective patient care [1, 2]. Globally, healthcare demands are increasing [3], and consequently, so is the need to educate healthcare professionals able to deliver safe and equitable care [4]. Clinical education research, defined here as research into the education, training and careers of healthcare professionals, plays a critical role in guiding how educational practice adapts to these challenges. However, this field of research is itself challenged by limited funding, which constrains its potential impact [5, 6].

*Research into the education, training and careers of healthcare professionals plays a critical role in guiding how educational practice adapts*.

Given these constraints, ensuring that research is appropriately focused on areas of need is key to maximising impact—beneficial change for healthcare education systems, learners, healthcare professionals and patients. A priority setting exercise, to identify current areas of need, is therefore desirable.

While priority setting exercises are recognised to be time‐dependent and risk being limited to ‘known’ needs, they can play a vital role in aligning resource allocation with the needs of multiple stakeholders [7, 8, 9]. Previous studies in clinical education research have explored priorities across the world [10, 11, 12, 13, 14], and despite the breadth of healthcare systems and geographies involved, have found several commonalities including the development of a sustainable workforce, ensuring health professionals' preparation for practice, their wellbeing, and continuing professional education.

The most recent UK‐based study, in 2014, considered the views of trainers and managers, patients and learners [11]. This reported that prioritisation varied between stakeholder groups: patients valued teamwork, interprofessional working and communication skills, while trainers and managers prioritised selection processes for medicine and faculty development. Learners prioritised research on balancing training and service, and effective learning cultures.

While research priorities will be determined in part by the context of a healthcare system and research economy, some of the factors that shape them are global. Advances in technology [15], changing professional roles [16] and the health needs of an ageing population [17] are common to many settings internationally, potentially shaping learners' clinical experience. In the UK, there have been substantial increases in the numbers of healthcare professionals in training [4], with potential implications for workplace learning and supervision. It is therefore timely to reassess research priorities to inform the future direction of clinical education research.

Given the critical role that shared priorities have in aligning resources and focusing research, we identified a gap in current knowledge, which can be addressed by an updated national prioritisation exercise for clinical education research. With awareness of the changed context since the most recent UK‐based study [11], our study also aimed to consider how different stakeholder groups approach prioritisation, something that has not previously been done. We considered three research questions:

What priorities for clinical education research activity are identified by stakeholders?

To what extent is there consensus about priorities?

How do stakeholders approach the question of prioritisation, and how does this relate to impact?

Together, these research questions will allow us to identify stakeholders' priorities, how they may vary and importantly, provide understanding of how the system may adapt as priorities change.

The work was carried out by the National Institute for Health Research (NIHR) funded Incubator for Clinical Education Research, which aims to increase capacity and visibility of the field in the United Kingdom [18]. The Incubator supports inclusive education research across all clinical professions, and so unlike earlier studies [10, 11, 12, 13, 14], we attempted to ensure representation across professions and stakeholder groups. We engaged funders and regulators, whose priorities may differ from those conducting research. Incorporating these viewpoints is important. If the priorities of those who are funding research, or using research to develop policy, are not aligned with those of researchers, there is a risk that opportunities for timely, impactful research are not realised.

*If priorities … are not aligned … there is a risk that opportunities … are not realised*.

## 2 Method

Research questions 1 and 2 were addressed using methods derived from established approaches to consensus building and prioritisation studies [19, 20]. This involved three stages:

1. A free‐text questionnaire to identify issues.
2. A quantitative questionnaire to identify priorities and examine consensus.
3. A hybrid (in person and online) workshop to discuss the process and parameters of prioritisation.

While using methodology derived from Delphi‐type studies, we did not seek to achieve consensus, or to simply rank priorities. Rather, the study aimed more expansively to explore the breadth of perspectives across participant groups. Rather than simply providing a list of desirable research, this approach allowed us to develop a more nuanced understanding of prioritisation.

## 3 Participants

Our sampling strategy aimed to include representative voices from a diverse range of stakeholder groups within and served by clinical education in the UK. We did not limit participation to recognised ‘experts’, so as not to privilege certain groups or experience, instead recognising that different perspectives bring their own expertise. Limiting our interest to those already working within clinical education research would provide only a partial view, biased by knowledge of current activity, and personal research interests. These are valid reasons for prioritisation, but limited. Our target populations therefore included researchers, educators, members of the public and representatives of stakeholder organisations. Participant groups and initial contacts to cascade invitations were identified through discussion within the Incubator and targeted conversations with some stakeholder groups (including the UK General Medical Council and Medical Protection Society).

### 3.1 Online Survey

We followed established methods [19, 20], using two rounds of surveys, firstly to gather the breadth of opinion amongst stakeholders, and secondly to quantify the extent of agreement with a set of priority statements derived from the first round. Both surveys were iteratively developed through engagement with the incubator for clinical education research and through working with a public contributor (author CH) to ensure clarity, content validity and acceptability.

#### 3.1.1 Round 1 Survey Content

The first survey gathered suggestions of priorities for clinical education research using free‐text questions. On the recommendation of an Incubator public partner (CH), two versions of the survey were used: one for those familiar with clinical education or education research (‘professionals’) and one for lay members of the public. Both surveys provided definitions of clinical education research, tailored to those audiences, to ensure that suggestions were appropriate.

The ‘professional’ survey asked participants to list topics they considered to be areas of unmet need in clinical education research. Additional prompts were provided to encourage respondents to consider a breadth of topics: ‘Getting the right people’, ‘Ensuring effective learning’, ‘Developing Professional Values’, ‘Assessing and Ensuring competence’, ‘Fairness/Equality’ and ‘Maintaining an effective workforce’. This survey also asked for respondents' organisational and professional background.

The public survey contained a single prompt asking for ‘suggestions for topics which you think are important for improving the education and training of healthcare professions, so that your experience as a patient would be improved’. Author CH advised on wording and clarity, to ensure a broad constituency of respondents would recognise the aims of the study. It also contained demographic items to monitor diversity.

The surveys were hosted on the JISC survey platform (https://app.onlinesurveys.jisc.ac.uk/). Surveys were pretested with individual public contributors and professionals to ensure clarity and content validity. Both surveys are provided in Data S1.

#### 3.1.2 Round 1 Analysis

The free‐text responses were summarised and synthesised by authors PY and BB through a process of content analysis and synthesis used in an earlier modified Delphi study [19]. They independently read all free‐text statements (approximately 1800 responses), coding statements into potential categories. Some of these categories were deductively informed by prior knowledge, and topics identified in the development of the survey, others were inductively identified in the data. The researchers then discussed and resolved any ambiguities in categorisation.

Statements within each category were then sorted, simplified, and iteratively reworded to provide simple statements of distinct areas of priority. This simplified list was reviewed by the wider team to ensure clarity, including input from a public contributor (CH) on readability for a public audience. This resulted in a list of 46 statements, which were used in Round 2.

During this process, statements were judged for relevance to the research questions, including consideration of scope and granularity. For example, very focused statements about individual conditions were felt to be too specific for a wider constituency of respondents to rate, and very broad statements were too general to constitute a meaningful priority.

#### 3.1.3 Round 2 Survey Content

A single version of the Round 2 survey was used, with wording reviewed by CH to ensure clarity, and distributed to all respondents. This allowed data from ‘professionals’ and ‘public’ to be analysed together.

Respondents were asked to rate the perceived importance of each of the statements derived from Round 1 on a 7‐point scale (with textual anchors at the extremes, ‘Not at all important: research in this area should not be funded’ and ‘Extremely important: research in this area should definitely be funded’). Rating, rather than ranking, items was judged to provide more credible data, more aligned with our research questions, as well as providing less burden on respondents (ranking even a subset of items would be onerous). A 7‐point scale was chosen for similar reasons, to allow respondents to moderate their level of agreement that a statement was or was not a priority.

We judged that operationalising priority in these terms provided a more concrete focus that the simple term ‘importance’ used in Round 1. All items included a response option, ‘This statement is not clear to me, or I do not know enough about the area’, to increase the credibility of responses. The organisation and individual role items from the expert Round 1 survey were also included.

The survey was sent directly to those who had provided an email address in response to Round 1. However, invitations were also cascaded through the same networks as Round 1, and further snowballing was encouraged. This was intended to reduce anchoring (respondents simply agreeing with their previously expressed priorities) and ensure a plurality of views.

#### 3.1.4 Round 2 Analysis

Following examination of distributions for skewness, descriptive statistics (median and inter‐quartile range) were calculated for ratings of each statement, to allow the ranking of items, and consideration of the diversity of opinion.

As many respondents selected two or more roles and organisations, it was not possible to reliably categorise respondents by single, nonoverlapping groups. We were, however, able to categorise respondents as ‘public’ or ‘professional’ to compare the rating of priority statements between these two groups by nonparametric test to address research questions 1 and 2. Analysis was undertaken in R v4.4.2 [21].

#### 3.1.5 Survey Distribution

Invitations to the Round 1 survey were distributed by email to the Incubator's established network and other contacts. Using the snowball method, these contacts were asked to cascade the study invitation to colleagues and other interested stakeholders. Invitations made it clear that the intent was to reach all involved in the sector, not just ‘experts’. Periodic reminders were sent to encourage responses. The first round survey was open for 42 days in June–July 2022, and the second round survey for 41 days in August–September.

### 3.2 Hybrid Workshop

Research question 3 was addressed in a hybrid (in person and online) workshop held in February 2023. Participants were invited through the same routes as the two surveys, and attendance was not limited to those who had responded to surveys, to ensure plurality of views. Participants were organised into 10 groups (seven in person and three online), each containing a mixture of stakeholders from different groups (policy makers, funders, patients, trainees, educational researchers and clinical educators) and a researcher to facilitate.

Groups were provided with a copy of the Round 2 statements, and asked to consider how they would rank the statements, and to articulate the values they used to assign priorities. These discussions were then reported to the wider workshop, which discussed each group's approach and identified points of agreement, disagreement and any principles for prioritisation. A second discussion considered how priority areas of research could best achieve impact.

Whole group discussions were audio‐recorded and transcribed. All authors independently read the transcripts and noted organising concepts within the data. Each small group facilitator made notes about discussions and identified key themes. These were collated by one researcher (PY) and then iteratively revised by all authors to produce a narrative overview.

### 3.3 Ethics and Governance

Newcastle University Research Ethics Committee deemed the study to be low risk (ref 21101), and full review was not required. Nonetheless, we ensured good ethical practice, in the wording of items, the security of data and gaining of informed consent for recording of workshops. All data were anonymised and stored on secure university systems.

## 4 Results

Tables 1 and 2 summarise respondents to the two surveys, categorised by organisational background and reported roles. Questions allowed multiple responses to be selected, meaning that rows do not sum to the total number of respondents.

**TABLE 1:** Reported organisation of respondents in survey Rounds 1 and 2.

Organisation	Round 1 (professional)	Round 1 (public)	Round 2
Total	115	141	199
Responding as individual	6 (5%)		64 (32%)
Patient organisation^a^	8 (7%)	70 (50%)	19 (10%)
Medicine	58 (50%)		74 (37%)
Dentistry	14 (12%)		14 (7%)
Nursing/midwifery	15 (13%)		7 (4%)
Pharmacy	15 (13%)		5 (3%)
Other allied health professional (including physiotherapy, physician associate and dietetics)	15 (13%)		13 (7%)
Regulator	2 (2%)		6 (3%)
Professional body	9 (8%)		11 (6%)
Funder	2 (2%)		2 (1%)
Higher Education Institutions (HEIs)	50 (43%)		64 (32%)
Other	8 (7%)		13 (7%)

*Note:* Responses to question: ‘Which of the following best describe the organisation(s) or group(s) experience on which you are basing your responses?’ Participants could select multiple options, meaning rows may not sum to the total.

^a^ In Round 1, respondents to the patient survey were asked, ‘Are you a member of or contributor to a patient group or organisation?’

**TABLE 2:** Reported role of respondents in survey Rounds 1 and 2.

Role	Round 1 (professional)	Round 1 (public)	Round 2
Total	115	141	199
Patient or member of the public	15 (13%)	141^a^	79 (40%)
Undergraduate/pre‐registration healthcare professional student	13 (11%)		10 (5%)
Postgraduate trainee in a healthcare profession	15 (13%)		16 (8%)
Doctoral student	10 (9%)		9 (4%)
Clinical teacher (clinician delivering education or training in the workplace)	48 (42%)		37 (18%)
Academic teacher/researcher with research interest in clinical education research	73 (63%)		72 (36%)
Policy lead for professional or regulatory body	7 (6%)		10 (5%)
Funding panel chair/member	6 (5%)		4 (2%)
Educational lead in a higher education institution (e.g., head of school)	30 (27%)		32 (16%)
Other	10 (9%)		18 (9%)

*Note:* Responses to question: ‘Which of the following describe your role(s) in relation to clinical education?’ Participants could select multiple options, meaning rows may not sum to the total.

^a^ No explicit question was asked in the public survey in Round 1.

The Round 1 survey was completed by 115 professional respondents and 128 public respondents (15 respondents to the professional survey also indicated they were responding as patients/public). It elicited 1819 individual text responses (1263 from the professional survey, 556 from the public survey), which were simplified and synthesised to 46 items used in Round 2.

There were 200 responses to Round 2, but one was removed as they had not rated any of the statements. Seventy‐nine (40%) of the remainder indicated that they were patients or members of the public, and 10 of these were representing a patient organisation. Due to the snowball cascade, we do not have denominators to calculate response rates to the survey, nor can we interpret the low numbers of responses from different groups—although some, such as funders, constitute a smaller population.

Eighty‐two people attended the workshop—17 online and 65 in person. Attendees included four patient/public representatives.

### 4.1 Round 2 Analysis: Respondent Priorities

The median of all but two items was above the scale midpoint of 4, while the lower quartile for 20 items was also above the midpoint. All items can therefore be seen as ‘important’ (summary statistics for all items are available in Data S1). Distributions of ratings did though indicate some variability of opinions, and the extent of agreement between respondents may indicate shared priorities. Table 3 shows the four items rated as important by at least 80% of respondents, indicating they are near‐universal priorities. These reflect an interesting mix of workforce sustainability and patient‐focused topics.

**TABLE 3:** Statements for which over 80% of responses were above the midpoint, indicating perceived importance.

Item text	Median importance (quartiles)	Frequency (%) of responses above the midpoint^a^	Frequency (%) of responses below the midpoint^a^
	Scale range 1–7		
Research to understand how working conditions and workplace cultures contribute to under‐recruitment in some roles and areas.	6 (5 to 7)	172 (86%)	17 (9%)
Research into the future sustainability of the clinical workforce (i.e., to understand why people join and leave)	7 (6 to 7)	170 (85%)	16 (8%)
Research to support a workplace culture, which promotes patient safety.	7 (5 to 7)	165 (83%)	18 (9%)
Research to ensure that clinicians are trained to effectively treat a diverse and evolving patient population.	6 (5 to 7)	164 (82%)	20 (10%)

^a^ Percentages of responses above and below the midpoint do not sum to 100% as statements at the midpoint did not contribute to either count.

Most respondents used a range of the scale across all items, indicating they considered items individually, rather than with a blanket judgment, or simply responding without consideration. There was a range of at least four scale points between the highest and lowest responses of 146 (74%) respondents. Just seven participants scored within a two‐point range across all items. This adds support for the survey's construct validity.

### 4.2 Differences Between Groups

Also of interest were differences in importance between ‘professional’ and ‘public’ stakeholders. For this analysis, those who indicated their only role was ‘patient or member of the public’, with no other options selected, were coded as ‘public’ (*n* = 67); all others were coded as ‘professional’ (*n* = 132).

Wilcoxon tests identified six statements where rankings of patients/public and professional respondents differed (summarised in Table 4. A Bonferroni correction was applied, so that 95% significance was indicated by *p* < 0.001). Significant differences on three items indicate that patients and members of the public rated research into communication skills and patient‐centred care as more worthy of funding than did professionals. One item showed public respondents rated research into the maintenance and assessment of skills more highly than professionals. The remaining significant difference indicated that research into new approaches to workplace learning was rated more highly by professionals than by patients.

**TABLE 4:** Items with significant differences between patient/public and professional ratings of item importance.

Item text	Public median (quartiles)	Professional mean median (quartiles)	Wilcoxon test value (*p*‐value^a^)
Research into clinicians' ability to communicate difficult ideas to patients with dignity and respect.	7 (6 to 7)	5 (4 to 5)	6055 (*p* < 0.0001)
Research into clinicians' ability to communicate effectively and sensitively with all patients and their families.	7 (6 to 7)	6 (4 to 6)	5990.5 (*p* < 0.0001)
Research into clinicians' ability to deliver patient‐centred care, which treats the whole person.	7 (6 to 7)	6 (4 to 6)	5674.5 (*p* < 0.0001)
Research into effective communication between clinicians and between different health and care services.	6 (5 to 6)	5 (4 to 5)	5626.5 (*p* = 0.0006)
Research into how clinicians' knowledge and skills are appropriately maintained and assessed throughout their careers.	7 (6 to 7)	6 (5 to 6)	5320 (*p* = 0.0006)
Research into novel approaches to workplace learning and supervision, including new placement structures.	5 (3.75 to 5)	6 (5 to 6)	2890.5 (*p* = 0.0003)

^a^ Difference is significant at 5% level, with Bonferroni correction applied.

### 4.3 What Is Prioritisation Based Upon?

The considerations behind prioritisation were discussed at the hybrid workshop, attended by over 80 participants from different stakeholder groups. Participants collectively agreed with the survey findings that most statements were important. They noted that some statements reflected perennial concerns (i.e., communication skills, feedback and novel approaches to learning), while others were potentially more acute.

Participants indicated that it would be not just difficult, but actively undesirable, to pick a ‘top ten’ list of statements, because importance is multifaceted, dependent on context, viewpoint and values.

So you know, to me a schematic just cut off Top 10 is not helpful … it's [a] useful foundation [to] the deliberative process … these are all helpful things, … but there's also space for research teams to come up with good ideas.[participant in whole‐group discussion]

They recognised that priorities differ between different stakeholder perspectives, and change over time. For example, the salience of some statements in the survey (notably the COVID‐related item) had lessened, and of others (particularly workforce issues) had increased at the time of the workshop.

Participants felt that prioritisation is inherently dynamic and value‐laden, and future knowledge needs are unpredictable.

It's necessary to build into a strategy, something that allows you flexibility over time to not only to respond to what's happening, but also to be able to build in a new … ways of addressing things.[participant in whole‐group discussion]

While bearing this in mind, they noted some priority areas, which are likely to require more focus over the medium term, including preparing the workforce for future needs, ensuring their continuing professional development to support wellbeing and recruitment and retention and the impact of developing technologies such as artificial intelligence. Participants also agreed that the meaningful impact and benefit to care of clinical education research can be direct, through enhancement of care or outcomes, or indirect, through changes to educational practice, which may enhance care or outcomes in the longer term.

You know, [a] discrete feasible research project that's gonna lead to a measurable patient impact. And you know, obviously how you measure patient impact is very debatable.[participant in whole‐group discussion]

Participants felt there is a balance to be maintained between short‐term priorities with immediate relevance and predictable impact, and strategic work to develop a knowledge base allowing responsiveness to future changes. This balance may be subject to potentially competing values held by different stakeholders, values which may also influence which topics should be prioritised. The difference between professional and public ratings of some items discussed in the previous section may indicate this difference in values, and in what is valued.

Participants discussed processes required to achieve impact, highlighting the importance of effective communication between stakeholders to recognise their different needs, priorities and values. Mechanisms to allow these to be shared and recognised will be important to a sustainable research culture.

## 5 Discussion

Over two rounds of online surveys, we identified 46 topics, which could constitute priorities for future research. Those which demonstrated the most consensus reflected recognition of the importance of both the workforce and patient experience in clinical education research, but all were rated as ‘important’ and deserving of funding. This highlights a key challenge in determining priorities, and leads us to consider what lies behind prioritisation, and how we can address this in policy and practice.

There were differences in the prioritisation of some topics by public and professional responses, reflecting their different viewpoints and values. Those of the public indicated concern with communication skills, clinicians' interactions with patients and the maintenance of skills, rather than the process of education and training. This has implications for how some priorities, which may be less clearly patient‐facing, such as curriculum integration or educational management, may be perceived by the public, and so how funders and researchers frame and justify some areas of research. Nonetheless, such topics may be examples of strategic, long‐term priorities.

*Public [representatives] indicated concern with communication skills, clinicians' interactions with patients, and the maintenance of skills*.

With input from over 200 respondents, including over 100 members of the public, our findings have credibility as being of wide relevance. However, while informative about perceived need, they highlight that making decisions about the funding of future research requires a collaborative and pragmatic approach informed by different stakeholder perspectives.

Compared with the most recent similar UK study [11], some concepts have remained important: workforce, wellbeing, assessment and feedback. However, issues are less important (e.g., curriculum integration), whilst others have evolved (simulation now encompasses virtual reality), grown (workforce issues being particularly salient in the UK) or emerged (artificial intelligence and machine learning). Our findings demonstrate a greater awareness of the importance of equality, diversity and inclusion. Comparison with international examples [10, 12, 13, 14] shows some similarity (the importance of preparedness and wellbeing), but also, the importance of national contexts (a focus on indigenous peoples in some countries, compared to the UK's interest in new clinical professions).

Our process benefitted from enabling people from a range of professional roles and experiences to work together. Despite this diversity, there was remarkable agreement across many priorities, although we have also illustrated some differences between professional and public views. Our workshop highlighted the contextual and dynamic nature of priorities; since the last UK prioritisation exercise [11], some topics have increased in priority, some have decreased and some (such as the COVID‐19 pandemic) had a prominence that is nonetheless transitory. These priorities should therefore not be seen as static, but rather serve as a reminder that the processes and principles that lead to priorities should be understood to ensure research meets both known and as‐yet unknown challenges.

*Priorities should therefore not be seen as static … the processes and principles which lead to priorities should be understood*.

### 5.1 Achieving Impact

An applied field of research such as clinical education should aim to achieve impact in practice by enhancing the quality, performance or capability of the healthcare workforce [22]. If different stakeholders have different priorities or do not recognise the reasons for others' priorities, then impact may be slowed.

We identified a distinction made by some between discrete impact, which may be demonstrated in the short term and more strategic, but potentially uncertain, impact in the longer term, and a potential tension between these considerations of impact. There is a risk that the longer‐term, more diffuse impact of education research is under‐recognised, and that there is a lack of an equivalent to ‘bench‐to‐bedside’ translational research.

*There is a risk that the longer‐term, more diffuse impact of education research is under‐recognised*.

At a practical level, our participants highlighted the importance to achieving impact of effective communication. A shared understanding of research priorities should act as the central focus of a network of stakeholders, to ensure that impact—whether short or long‐term—is maximised in practice.

**FIGURE 1:**
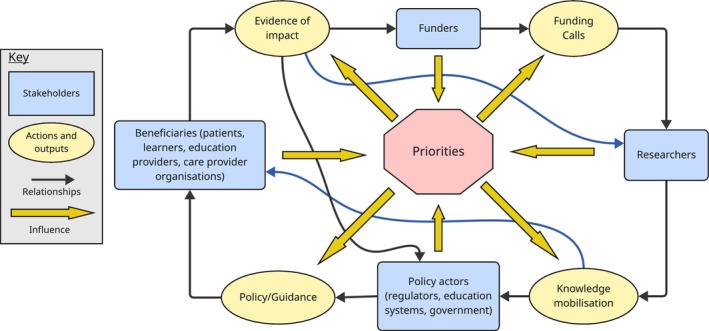
Illustration of the ‘System of Impact’, and relationships between different stakeholders.

From our findings, and discussions within the incubator for clinical education research, we developed a conceptual model of a proposed impact network (Figure 1). This illustrates the various stakeholders within this network and key relationships between them. Each may initiate research ideas, but their ‘power’ to develop projects, and to achieve impact or change, varies. Recognising, and nurturing, an integrated network to support this ‘system of impact’ throughout the research process (e.g., initial patient engagement during research proposal development, through to stakeholder network engagement at dissemination stages) can maximise the relevance and potential impact of research approaches and outputs.

The key relationships in this system are the following:

**Funders—researchers**: Effective communication here can ensure panels are sensitised to the language, theory and methodologies most suited for projects in priority areas and are aware of the breadth of approaches, which allow issues to be explored in a variety of ways and from different perspectives. Researchers can be reassured their proposals are meeting the need.
**Researchers—policy actors**: Knowledge mobilisation to policy stakeholders, including regulators and government bodies, is essential to ensure evidence is clearly received and understood by those stakeholders, enabling direct and indirect impact. Collaboration with these stakeholders within the network can also ensure the feasibility and evaluability of change.
**Policy actors and researchers—beneficiaries**: Effective communication of intended impact, from researchers and from policy actors, will ensure that the intended beneficiaries of the research (including patients, learners and educators) are equal partners in implementation, enabling changes in practice to take place.
**Beneficiaries and policy actors—funders**: Closing this system are relationships between knowledge users and funders to guide and inform what research should be funded due to immediate or longer‐term need. Beneficiaries can feed back on what is or is not working at a local level, and inform further research needs.

### 5.2 Recommendations for Practice

Our key recommendation for practice, building from these concepts, is that all stakeholders should recognise the existence and benefits of, and challenges to, effective relationships within the system of impact, and collectively consider ways in which it can be optimised. At present, effective communication may occur at a project or programme level, where researchers, funders, public and patient representatives and other stakeholders consider individual studies and findings, but we should aspire to it becoming an embedded cultural norm across the research landscape.

*All stakeholders should recognise the existence and benefits of, and challenges to, effective relationships within the system of impact*.

Actions to support this may include regular communication between stakeholders to coordinate and maximise dissemination. Pragmatically, communication and coordination may be achieved through cross‐disciplinary events such as our in‐person workshop, but a shared strategic approach to knowledge mobilisation encompassing targeted channels of events, reports and may be more effective for reaching different audiences across the system.

Direction may be found in work on stakeholder engagement in other health‐related disciplines [23, 24], where many stakeholders will be the same as in clinical education research, even if the aims and nature of impact may be distinct. Future work may consider how these approaches translate to clinical education research.

### 5.3 Limitations

Whilst the process followed in this study was highly dependable, all research has limitations. Firstly, we only gathered data in a UK context. In noting that priorities are contextual, we acknowledge limits to the transferability of any consideration of priority, but the principles we have described are likely to resonate in other contexts. Where many healthcare concerns are faced on a global scale—disease, demographics and resources—recognising common ground, as well as contextual difference, is important.

We engaged a broad range of stakeholders, but recognise that a different sample may have identified different or additional topics or concerns, based on their existing knowledge of, and engagement with, clinical education research. Our sample did not include politicians, and the political and policy drivers for change and research activity are perhaps the elephants in the room when considering what will, rather than what should be, funded. Nonetheless, the involvement of multiple stakeholder groups and the inclusion of additional individuals during each phase of our methods helps to assure that we reached a genuine cross‐section of the UK clinical education population. While we deliberately chose not to collect demographic data from professionals, as they were intended to respond ‘ex officio’ rather than individually, the absence of this information does limit conclusions about sampling bias.

Our operationalisation of importance in our second survey did not allow us to identify specific priorities, but it did not aim to. A different operationalisation of importance, for example, focusing on resource allocation, may have allowed more specific identification of priorities, but our chosen approach illuminates the challenges and compromises of such pragmatism, and that ‘important’ research will always go unfunded.

In considering the rankings of different groups, we distinguished between ‘professional’ and ‘public’ contributors. We accept that this grouping was simplistic, but opted for this approach as many professionals held multiple designations making it impossible to discretely categorize them. Future research might determine whether, and how, priorities vary between the perspectives of different professional roles.

## 6 Conclusions

Working with relevant stakeholder groups, we have suggested broad priorities for clinical education research in the United Kingdom, but recognise that simple prioritisation is a crude approach and may vary with stakeholder perspectives and immediate contexts. We have therefore also developed an understanding of how priorities arise, including recognising and responding to differences between stakeholder groups, changes over time, and perceptions and value of impact. A recognition that the impact of research may be deferred is an important part of developing an effective system. Funding opportunities should be agile in their scope and focus, and policy actors remain alert to a need for rigorous evidence to inform policy changes (in workforce, education and training).

We propose that recognising a joined‐up ‘system of impact’ can support future research to maximise opportunities for stakeholders—researchers, health care professionals, educators, patients, public and policymakers—to collaborate, exchange ideas and effectively communicate [25]. Embedding ongoing exchange of knowledge and needs in the system of research impact will optimise that impact in the short and longer term.

*Recognising a joined‐up ‘system of impact’ can support future research*.

## Author Contributions

**Bryan Burford:** conceptualization, investigation, writing – original draft, methodology, writing – review and editing, formal analysis, project administration. **Peter Yeates:** conceptualization, investigation, writing – original draft, methodology, writing – review and editing, formal analysis. **Anna Harvey Bluemel:** investigation, writing – original draft, writing – review and editing, formal analysis. **Sophie Park:** conceptualization, methodology, writing – review and editing, formal analysis, investigation. **John Sandars:** conceptualization, methodology, formal analysis, investigation, writing – review and editing. **Cecily Henry:** conceptualization, methodology, investigation, formal analysis, writing – review and editing. **Clare Corness‐Parr:** investigation, formal analysis, writing – review and editing. **Richard Conn:** conceptualization, methodology, investigation, formal analysis, writing – review and editing. **Tom Gale:** investigation, formal analysis, writing – review and editing. **Tim O'Brien:** conceptualization, methodology, investigation, formal analysis, writing – review and editing. **Rikki Goddard‐Fuller:** investigation, formal analysis, writing – review and editing. **Gill Vance:** conceptualization, methodology, investigation, formal analysis, writing – review and editing. **Janice Ellis:** conceptualization, methodology, investigation, formal analysis, writing – review and editing.

## Ethics Statement

Ethical approval was received from Newcastle University Research Ethics Committee (ref 21101).

## Conflicts of Interest

The authors declare no conflicts of interest.

## Supporting information


**Data S1** Descriptive statistics for all Round 2 statements, sorted by decreasing frequency of responses above the midpoint on importance scale.

## Data Availability

Quantitative data is available on reasonable request by contacting the corresponding author.
