# Supplementary material for: Establishing Priorities for Clinical Education Research: Exploring the Views of UK Professional and Public Stakeholders
Source: Clin Teach. 2025 Jun 30;22(4):e70144. doi: 10.1111/tct.70144 (PMC12209556; doi:10.1111/tct.70144)
Supplement: Supplementary file 1 — Data S1 Descriptive statistics for all Round 2 statements, sorted by decreasing frequency of responses above the midpoint on importance scale. [file TCT-22-e70144-s001.docx]

Supplementary material 1. Descriptive statistics for all Round 2 statements, sorted by decreasing frequency of responses above the midpoint on importance scale.

| **Item text** | **Median importance (quartiles)**  **Scale range 1-7** | **Frequency (%) of responses above the midpoint** | **Frequency (%) of responses below the midpoint** |
| --- | --- | --- | --- |
| Research to understand how working conditions and workplace cultures contribute to under-recruitment in some roles and areas. | 6 (5 to 7%) | 172 (86%) | 17 (8%) |
| Research into the future sustainability of the clinical workforce (ie to understand why people join and leave) | 7 (6 to 7%) | 170 (85%) | 16 (8%) |
| Research to support a workplace culture which promotes patient safety. | 7 (5 to 7%) | 165 (82%) | 18 (9%) |
| Research to ensure that clinicians are trained to effectively treat a diverse and evolving patient population. | 6 (5 to 7%) | 164 (82%) | 20 (10%) |
| Research into how clinicians' knowledge and skills are appropriately maintained and assessed throughout their careers. | 6 (5 to 7%) | 158 (79%) | 18 (9%) |
| Research into how new technologies (such as simulation and virtual reality) may be best used in clinical education. | 6 (5 to 7%) | 156 (78%) | 21 (10%) |
| Research to reduce or eradicate inappropriate workplace behaviour including bullying, undermining, discrimination and harassment. | 6 (5 to 7%) | 155 (78%) | 22 (11%) |
| Research into how workplaces can provide effective learning environments. | 6 (5 to 7%) | 153 (76%) | 24 (12%) |
| Research into processes to select, progress and sustain a wider, more diverse, workforce. | 6 (5 to 7%) | 153 (76%) | 26 (13%) |
| Research into clinicians' ability to deliver patient-centred care which treats the whole person. | 6 (5 to 7%) | 153 (76%) | 21 (10%) |
| Research into how new and changing clinical roles will shape the future workforce. | 6 (5 to 7%) | 152 (76%) | 16 (8%) |
| Research into clinicians' ability to communicate effectively and sensitively with all patients and their families. | 6 (5 to 7%) | 152 (76%) | 24 (12%) |
| Research into clinicians' ability to communicate difficult ideas to patients with dignity and respect. | 6 (5 to 7%) | 152 (76%) | 24 (12%) |
| Research into novel approaches to workplace learning and supervision, including new placement structures. | 6 (5 to 6%) | 151 (76%) | 24 (12%) |
| Research into effective communication between clinicians and between different health and care services. | 6 (5 to 7%) | 150 (75%) | 23 (12%) |
| Research into the effectiveness of workplace-based assessment/eportfolio approaches. | 6 (5 to 7%) | 148 (74%) | 24 (12%) |
| Research into how the cultures of healthcare organisations supports staff wellbeing. | 6 (4.25 to 7%) | 148 (74%) | 29 (14%) |
| Research to ensure that feedback effectively supports students' and trainees' learning. | 6 (4.5 to 7%) | 146 (73%) | 27 (14%) |
| Research into how technological changes such as the internet / machine learning are changing how people learn and practice. | 6 (4 to 6%) | 146 (73%) | 24 (12%) |
| Research to understand the implications of initiatives to widen access to health professions for long term workforce and patient experience. | 6 (5 to 7%) | 146 (73%) | 28 (14%) |
| Research to understand and eliminate systemic, overt and unconscious bias including microaggressions from training environments. | 6 (4.75 to 7%) | 144 (72%) | 33 (16%) |
| Research to ensure assessments can identify areas of relative strength and weakness in learners' performance. | 6 (4 to 6%) | 141 (70%) | 30 (15%) |
| Research to ensure that clinical education can cope with increased student numbers. | 5 (4 to 6%) | 141 (70%) | 36 (18%) |
| Research to understand how learners learn, retain and apply knowledge to clinical practice. | 5 (4 to 6%) | 140 (70%) | 35 (18%) |
| Research to translate and apply theory from broader educational fields (i.e. psychology, sociology, neuroscience) to clinical education. | 6 (4 to 6.5%) | 140 (70%) | 23 (12%) |
| Research to ensure that career pathways are flexible and appropriate for a diverse population of staff. | 6 (4 to 6%) | 139 (70%) | 28 (14%) |
| Research to examine the effectiveness of programmatic or continuous assessment in clinical education. | 6 (4 to 6.25%) | 135 (68%) | 25 (12%) |
| Research to understand and eliminate differences in qualification and career destinations between different demographic groups. | 6 (4 to 7%) | 135 (68%) | 28 (14%) |
| Research into the value and effectiveness of patient involvement in selection, education, training and assessment. | 6 (4 to 7%) | 135 (68%) | 31 (16%) |
| Research into the individual and organisational factors which shape learner and staff wellbeing. | 6 (4 to 6%) | 134 (67%) | 36 (18%) |
| Research to support patients' understanding of the health service and professional roles within it. | 5 (4 to 6%) | 130 (65%) | 33 (16%) |
| Research to understand and support learner experiences as they transition between career stages. | 5 (4 to 6%) | 127 (64%) | 32 (16%) |
| Research into the ways in which different professions learn and work together. | 5 (4 to 6%) | 127 (64%) | 38 (19%) |
| Research into flexible training paths including part-time working. | 5 (4 to 6%) | 126 (63%) | 34 (17%) |
| Research linking education and training to patient outcomes through the use of large data sets. | 5.5 (4 to 7%) | 126 (63%) | 26 (13%) |
| Research into the development and operation of professionalism and professional identity and how these impact clinical practice. | 5 (4 to 7%) | 126 (63%) | 39 (20%) |
| Research to support clinicians' ability to provide generalist and holistic care. | 5 (4 to 6%) | 122 (61%) | 38 (19%) |
| Research into developing clinicians' readiness for leadership roles. | 5 (4 to 6%) | 120 (60%) | 33 (16%) |
| Research to explore and support the authenticity or realism of assessment in relation to clinical practice. | 5 (4 to 7%) | 119 (60%) | 24 (12%) |
| Research to ensure people are able to achieve careers that fit their abilities, temperaments, and preferences. | 5 (4 to 6%) | 118 (59%) | 40 (20%) |
| Research to identify and reduce threats to the robustness, security or validity of assessment. | 5 (4 to 6%) | 106 (53%) | 39 (20%) |
| Research into global healthcare education. | 5 (3 to 6%) | 102 (51%) | 52 (26%) |
| Research into the use of large data sets in the planning and delivery of education and training. | 5 (4 to 6%) | 100 (50%) | 37 (18%) |
| Research to establish how performance in formal assessments or selection tests is linked to people's later careers. | 5 (3 to 6%) | 99 (50%) | 53 (26%) |
| Research to understand the career choices clinicians make. | 4 (4 to 6%) | 98 (49%) | 47 (24%) |
| Research into the long-term impact of Covid on education, training and careers. | 4 (3 to 6%) | 91 (46%) | 63 (32%) |

* Percentages of responses above and below the midpoint do not sum to 100% as statements at the midpoint did not contribute to either count.
